# Treatment of a Complex Emulsion of a Surfactant with Chlorinated Organic Compounds from Lindane Wastes under Alkaline Conditions by Air Stripping

**DOI:** 10.1021/acs.iecr.2c03722

**Published:** 2023-02-07

**Authors:** Patricia Sáez, Raúl García-Cervilla, Aurora Santos, Arturo Romero, David Lorenzo

**Affiliations:** Chemical Engineering and Materials Department, Complutense University of Madrid, 28040 Madrid, Spain

## Abstract

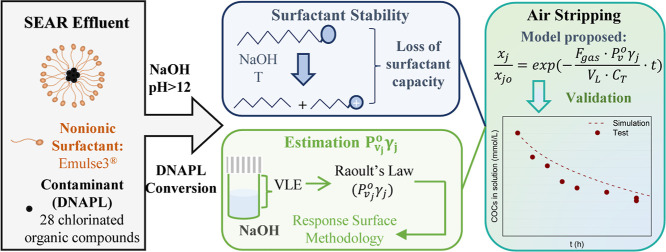

Surfactant-enhanced aquifer remediation is commonly applied in polluted sites with dense non-aqueous phase liquids (DNAPLs). This technique transfers the contamination from subsoil to an extracted emulsion, which requires further treatment. This work investigated the treatment of a complex emulsion composed of a nonionic surfactant and real DNAPL formed of chlorinated organic compounds (COCs) and generated as a lindane production waste by air stripping under alkaline conditions. The influence of the surfactant (1.5–15 g·L^–1^), COC concentrations (2.3–46.9 mmol·L^–1^), and temperature (30–60 °C) on the COC volatilization was studied and modeled in terms of an apparent constant of Henry at pH > 12. In addition, the surfactant stability was studied as a function of temperature (20–60 °C) and surfactant (2–10 g·L^–1^), COC (0–70.3 mmol·L^–1^), and NaOH (0–4 g·L^–1^) concentrations. A kinetic model was successfully proposed to explain the loss of surfactant capacity (SCL). The results showed that alkali and temperature caused the SCL by hydrolysis of the surfactant molecule. The increasing surfactant concentration decreased the COC volatility, whereas the temperature improved the COC volatilization. Finally, the volatilization of COCs in alkaline emulsions by air stripping (3 L·h^–1^) was performed to evaluate the treatment of an emulsion composed of the COCs (17.6 mmol·kg^–1^) and surfactant (3.5 and 7 g·L^–1^). The air stripping was successfully applied to remove COCs (>90%), reaching an SCL of 80% at 60 °C after 8 h. Volatilization can remove COCs from emulsions and break them, enhancing their further disposal.

## 1 Introduction

Soil and groundwater contamination by organic compounds has become a severe environmental issue.^1^ The accidental release or intentional dumping of hydrophobic organic liquid phases into the environment is a widespread problem resulting in a separate liquid phase, termed non-aqueous phase liquids (or NAPLs), that persists in the subsurface.^2^ Prolonged contact between soil and water with these NAPLs can impact the organisms of the food chain, harming human health and ecosystems.^3^ In the last decades, this contamination has been associated with pesticides, veterinary drugs,^4^ and heavy metals^5^ released as industrial wastes. These compounds can affect water bodies, producing significant problems like antibiotic resistance, sex organ imposition, and many others.^6^

An effective treatment to remediate polluted sites with NAPLs is the application of surfactant enhancement aquifer remediation (SEAR).^7^ This technique injects an aqueous solution containing a surfactant into the contaminated area. Then, a polluted stream composed of a mixture of organic compounds^8^ and the surfactant injected is extracted. This stream can be a mixture of Tween-80 and total petroleum hydrocarbons (TPHs)^9^ or tetrachloroethene-nonaqueous^10^ and chlorinated organic compounds (COCs) with E-Mulse 3 (E3).^11^

The surfactants enhance the removal of pollutants through solubilization and mobilization. The amphoteric properties of the surfactants that reduce interface tension facilitate the transport of hydrophobic pollutants to the aqueous phase.^12^ The SEAR technique presents significant benefits compared to other technologies, such as pump and treat,^11^ since it increases the rate of NAPL removal. However, the SEAR process moves the organic contamination into the aqueous phase but does not eliminate the contaminant, resulting in secondary contamination.^7^ A low soil permeability limits the applicability of the SEAR technology.^11^ The adsorption of the surfactants and a possible dispersion of contaminants from the control zone affect the efficiency and safety of the process.^13^

Once these disadvantages are overcome, the SEAR process can be applied successfully.^13^ It was reported that the use of a surfactant improves the elimination of TPHs about 75 times the amount removed with water alone^14^ or increased by 2 orders of magnitude the elimination of tetrachloroethene (PCE) using an aqueous solution of the 6% w Tween-80 surfactant.^10^ In addition, using E-Mulse 3 (E3) allowed the solubilization of COCs, removing about 3.5% of COCs in the soil using only a pore volume of the aqueous surfactant solution (effective porosity of soil is less than 0.12) after 15 h of injection treatment. In these applications, the emulsion extracted from the subsoil contained a complex mixture of organic compounds, and the surfactant used and this emulsion must be managed appropriately.

Several technologies have been proposed for this scope. Some papers consider the selective oxidation of organic compounds in the mixture with the recovery of the surfactant capacity. Hanafiah et al. applied ultrafiltration and permanganate to recover the surfactant used in the remediation of a site polluted with polycyclic aromatic hydrocarbons (PAHs).^15^ Huang et al. used ferric ions in the photo-treatment of Brij35 washing waste containing 2,2′,4,4′-tetrabromodiphenyl ether.^16^ Li et al. used electrochemically reversible foam-enhanced flushing for PAH-contaminated soil with FC12 as the surfactant.^17^ García-Cervilla et al. studied the compatibility of E3 and sodium dodecyl sulfate (SDS) with persulfate activated by alkali in the reduction of COCs.^18^ In these treatments, the COCs are not mineralized, and there is a loss of surfactant stability associated with the unproductive consumption of the oxidant by the surfactant.^18^

Selective adsorption of organic pollutants on activated carbon (AC)^19^ and selective retention by membranes^20^ have also been investigated in the remediation of SEAR streams.

The air stripping of COCs in the emulsion has been reported but scarcely studied in the literature. This technique transfers the volatile compound from an aqueous solution to an air stream, and it could be effective when the organic compounds are volatile or semivolatile.^21^ When the polluted emulsion is directly sent for adsorption on AC, the efficiency of the process remarkably decreases due to the quick saturation of AC with the surfactant.^19^ On the contrary, the transfer of the organic pollutants from the emulsion to an air stream, free of the surfactant, remarkably improves the efficiency and economy of the further pollutant adsorption on AC.

The volatility of organic compounds from the emulsion has been studied using an apparent Henry’s law constant to determine the vapor–liquid partitioning of chlorinated solvents in surfactant solutions.^22^ This apparent constant considers a three-phase system where the volatile organic compounds are partitioned into vapor, extramicellar (aqueous), and micellar phases.^23^ Some authors have studied the partition of pure compounds between vapor and emulsion phases. Shimtory et al.^24^ measured and estimated apparent Henry’s constants of pure compounds [TCE, PCE, *cis*-dichloroethylene (DCE), and *trans*-dichloroethylene] with different surfactants (SDS, Triton X-100, and bromuro de cetiltrimetilamonio). They found a dependence between the concentration and surfactant type.^24^ Similar conclusions were reported by Zhang et al.^25^ They tested three pure compounds (TCE, PCE, and DCE) separately using SDS, sodium dodecyl benzene sulfonate, Tween-80, and Triton X-100.^25^

In the same way, Sprunger et al. studied the partition between extramicellar and micellar phases and the volatilization of several pure compounds using SDS as a surfactant.^26^ Also, recently, Chao et al. used 1,2-dichlorobenzene (1,2-DCB), *1*,*3*,*5*-trichlorobenzene (1,3,5-TCB), and 1,2,3,4-tetrachlorobenzene (1,2,3,4-TetraCB) with different Triton surfactants and reported a relationship between the volatilization and solubility of this compound and surfactant type.^27^ In these studies, pure compounds were used as model compounds to study the COC volatilization. However, there is scarce information on using a complex mixture of COCs as wastes of pesticides such as lindane. In addition, E3 has not been previously studied as a biodegradable surfactant.

This work aims to study and model air stripping applications to volatilize COCs in an aqueous emulsion with nonionic and biodegradable surfactants. The emulsion used is a real effluent generated after a SEAR treatment of a polluted site in Sardas landfill (Sabiñánigo, Spain). In this place, the liquid wastes of lindane production, containing 28 COCs, were dumped in unlined landfills, migrating vertically through the aquifer as dense NAPLs (DNAPLs) and polluting the nearby area.^28^ In previous studies, it was reported that alkali addition could be considered to enhance the volatility of the more chlorinated compounds since it promotes their dehydrochlorination to more volatile compounds in the aqueous and soil phases^29^ and emulsion with E3, Tween-80, and SDS.^30^ This effect can be managed by air stripping the contaminated emulsions obtained after SEAR treatment of sites polluted with DNAPL waste from lindane production. However, the alkali, surfactant concentrations, and temperature could affect the surfactant stability and the volatility of the COCs in the emulsion. These aspects have not been previously studied for a complex organic phase in the literature but are required for a proper air-stripping treatment design. The latter required the study of the volatility of each COC in the alkaline emulsion and the surfactant stability at different alkali concentrations and temperatures. Predicted and experimental values during air stripping runs will also be compared to validate the model proposed and the parameters obtained.

## 2 Materials and Methods

### 2.1 Chemicals and DNAPLs

The quantification of COCs was performed using calibration curves prepared from commercial compounds (Sigma-Aldrich, analytical grade): chlorobenzene (CB), 1,2-DCB, 1,3-dichlorobenzene (1,3-DCB), 1,4-dichlorobenzene (1,4-DCB), 1,2,3-trichlorobenzene (1,2,3-TCB), 1,2,3,4-TetraCB, 1,2,3,5-tetrachlorobenzene (1,2,3,5-TetraCB), 1,2,3,4-tetrachlorobenzene (1,2,3,4-TetraCB), and hexachlorocyclohexane isomers (α, β, γ, δ, and ε-HCH). Additionally, limonene [(R)-(+)-limonene, Sigma-Aldrich] (cosolvent of the surfactant) was also calibrated. Bicyclohexyl (C_12_H_22_, Sigma-Aldrich) and tetrachloroethane (C_2_H_2_Cl_4_, Sigma-Aldrich) were used as internal standards (ISTD) for quantification by gas chromatography (GC).

Two DNAPLs were used in this work. On the one hand, a real DNAPL (DNAPL-R) was obtained from a contaminated site in Sabiñánigo (Spain). The DNAPL-R samples were provided by the company Emgrisa and the Aragon Government. The composition of DNAPL-R used is summarized in Table S1 of the Supporting Information DNAPL-R is composed of 28 COCs: CB, the isomers of dichlorobenzene (lumped as DCBs), trichlorobenzene (lumped as TCBs), tetrachlorobenzene (lumped as TetraCBs), pentachlorolcyclohexenes (lumped as PentaCXs), hexachlorocyclohexane (lumped as HCHs), hexachlorocyclohexene (lumped as HexaCXs), and heptachlorocyclohexanes (lumped as HeptaCHs).

NaOH was used to promote the alkaline dehydrochlorination of HCHs and PentaCXs to TCBs and HeptaCHs and HexaCXs and HeptaCHs to TetraCBs, which reduced the toxicity of the effluent to be treated and increased the volatility of the COCs in the aqueous phase.^30^ The composition of DNAPL-R after the alkalinization treatment (i.e., xi pH > 12) is summarized in Table S1.

Additionally, a synthetic DNAPL (DNAPL-S) was used to simulate the COC composition of the real DNAPL-R due to the limited amount of DNAPL-R available after alkaline treatment. Commercial compounds (CB, 1,2-DCB, 1,2,4-TCB, 1,2,3 TCB, and a mixture of 1,2,4,5-tetrachlorobenzene, 1,2,3,5-TetraCB, and 1,2,3,4-TetraCB) were mixed to produce DNAPL-S. The molar fractions of these compounds in DNAPL-S are shown in Table S2.

The surfactant used to carry out the experiments was E-Mulse 3 (E3) (EthicalChem), which is a nonionic surfactant with a critical micelle concentration measure of 80 mg·L^–1^. E3 was selected because it is a biodegradable and non-toxic surfactant^11^ and has been successfully applied in the solubilization of COCs from DNAPL-R to the aqueous phase.^31^

The air employed to perform the experiments was supplied by Carburos Metálicos, with a quality of 99.999% . The aqueous solutions were prepared with high-purity water from a Millipore Direct-Q system with resistivity >18 mΩ·cm at 25 °C.

### 2.2 Experimental Procedure

The experimental procedure was divided into three experiment sets. In the first one (B1), the surfactant stability was studied at different temperatures and NaOH doses, and a kinetics for surfactant capacity loss (SCL) was obtained. The second procedure studied the volatilization of each chlorinated compound in the complex mixture of the surfactant and DNAPL-R (B2). In this set of experiments, different temperatures and surfactant and COC concentrations were established to study the amount of the volatile compound transferred to the vapor phase. Finally, volatilization of COCs in the emulsion (set B3) was carried out by passing an airstream through the aqueous emulsion at several surfactant concentrations and temperatures. During these experiments, the surfactant loss by the reaction was not renewed, and the surfactant load was added at the initial time.

#### 2.2.1 Surfactant Stability (B1)

Surfactant stability experiments were conducted in the batch mode using sealed GC 20 mL glass vials without headspace closed with Teflon caps in the absence and presence of COCs. In the last case, a certain amount of DNAPL-S was added to the aqueous phase with the surfactant (2–10 g·L^–1^) to obtain a stable emulsion. The moles of solubilized organic compounds per mole of the surfactant in micellar solution was the molar solubilization ratio (MSR) for DNAPL-R or DNAPL-S in E3, as determined elsewhere^31^ and resulting in 4.33 mmol COCs·g_surf_^–1^. The emulsions were agitated for 4 h and left to settle for 24 h without agitation; after this time, the concentration of COCs in the emulsion was stable over time, and the amount of COCs was measured. The emulsions prepared following this experimental procedure, in which the ratio of the mole DNAPL and surfactant corresponds with the MSR, will be identified as saturated emulsions in COCs.

The vials were prepared with 19 mL of surfactant solution (containing or not containing DNAPL-S). Then, the vials were heated in a thermostatic bath to obtain the desired temperatures (25–60 °C). Once the temperature was reached, 1 mL of NaOH was added (zero time) into the vials from a concentrated stored aqueous solution to obtain the required NaOH concentration in the vial (2 or 4 . It is important to point out that 2 g·L^–1^ is the minimum quantity of alkali required to get a total dehydrochlorination.^29^ A pH of 12 was obtained with this NaOH concentration. A magnetic stirrer continuously agitated the alkalized emulsion at the desired temperature. The experimental conditions are summarized in Table 1. The MSR and molar concentration appearing in Table 1 have been calculated using the averaged molecular weight obtained from the known composition of both DNAPLs used (DNAPL-S and DNAPL-R). In the case of DNAPL-R, its characterization was carried out in previous work.^28^ The average molecular weight of DNAPL-S is 164 g·mol^–1^ whereas for DNAPL-R is 196 g·mol^–1^.

**Table 1:** Experimental Conditions for the Three Experiment Sets

Exp	*T* (°C)	*C*_S0_ (g·L–^1^)	*C*_NaOH_ (g·L–^1^)	*C*_DNAPL_ (mmol·L^–^1)
B1: Surfactant Stability				
E1	20	10	2	0
E2	20	5	2	0
E3	20	10	4	0
E4	20	5	4	0
E5	20	2	4	0
E6	40	5	4	0
E7	60	10	2	0
E8	60	10	4	0
E9	60	5	4	0
E10	20	10	2	70.3
E11	20	2	2	14.6
E12	40	5	4	35.2
E13	60	10	2	70.3
E14	60	10	4	70.3
E15	60	5	4	35.2
E16	20	10	0	0
E17	60	10	0	0
B2: Estimation of the Apparent Henry’s Constant				
P1	30, 40, 60	1.5	5	2.3, 4.7
P2	30,40, 60	3.5	5	5.9, 14.6
P3	30, 40, 60	7.0	5	5.9, 17.6, 29.3
P4	30, 40, 60	15.0	5	11.7, 23.4, 46.9
B3: Volatilization Tests				
V1	40	3.5	4	17.6
V2	60	7.0	4	17.6

The remaining equivalent surfactant concentration (ESC) was analyzed by sacrificing a vial at different reaction times, including 0. In the experiments carried out with saturated emulsion of DNAPL-S, the remaining ESC at each time was calculated from the remaining COCs in solution, taking into account the MSR value, as shown in eq 1. In the absence of DNAPL, the remaining ESC at each time was calculated by dissolving 1,3 DCB and measuring the solubilized concentration of this compound in the emulsion phase . The concentration of the surfactant calculated using this method is an ESC, which considers the products and subproducts capable of dissolving COCs lumped as the surfactant. The experiments were replicated, finding a discrepancy between experimental results lower than 5%. The average values were used as the experimental results.
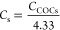
1where *C*_S_ is the ESC (g_surf_·L^–1^) and *C*_COCs_ is the concentration of the sum of COCs  and 4.33 is the MSR of E3 with the DNAPL-R and 13-DCB in mmol_COCs_·g_surf_^–1^.

#### 2.2.2 Estimation of Apparent Henry’s Constant (B2)

This set of experiments was carried out to obtain the apparent value of Henry’s constant of each COC (*j*) in the presence of the surfactant.

In 100 mL flasks, an amount of DNAPL-R (ranging from 0.04 to 0.8 g in order to get a concentration between 2.3 and 46.9 mmol·L^–1^) was added and filled up to 100 g with the corresponding aqueous phase containing the surfactant (E3 concentration ranging from 1.5 to 15 g·L^–1^). The amount of DNAPL-R added was always less than that required for saturation. After 2 h of agitation, the solution was settled, and DNAPL-R as an organic phase was not noticed. Following this, 10 mL of the emulsion was transferred to 20 mL GC glass vials, and NaOH was added to reach a concentration of 5 g·L^–1^ (pH > 12). Then, the vials were closed and agitated at different controlled temperatures (30–60 °C) for 1 h in the incubator of HeadSpace GC (HS-GC), Agilent GC Sampler 120. This time was enough to reach equilibrium between the liquid and vapor phases. The COCs in the vapor phase were analyzed by HeadSpace, following the methodology used elsewhere^32^ coupled with GC/ flame ionization detector (FID)/ electron capture detector (ECD). Table 1 summarizes the conditions of the experiments in set B2.

#### 2.2.3 Volatilization Tests (B3)

**Figure 1:**
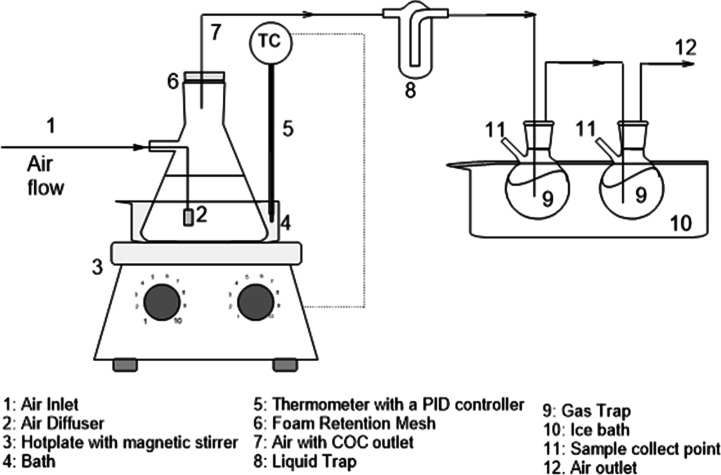
Scheme of the installation used for volatilization tests.

The volatilization of chlorinated compounds from the aqueous surfactant emulsion was carried out in the experimental setup schematized in Figure 1. An airstream was bubbled in the surfactant solution with solubilized DNAPL-S at pH > 12. The air was fed to the experimental system from pressurized air in a cylinder, and the flow rate was controlled using a mass flow controller (EL-FLOW Select Series Mass Flow Meters/Controllers for gases, Bronkhorst). A diffuser introduced the air into the emulsion to ensure a high interphase favoring the gas–liquid equilibrium achievement. The recipient containing the emulsion was immersed in a water bath placed on a hotplate (IKA C-MAG HS 7). The temperature was controlled using a PID controller (IKA ETS-D5) thermometer. After reaching liquid–gas equilibrium, the gas effluent saturated in COCs was passed through an iron mesh (100 μm) to prevent excessive foam formation and was bubbled in MeOH, which acted like a liquid trap. The MeOH traps were introduced into an ice bath to avoid volatile loss. Samples were taken periodically from the emulsion, the remaining COCs were analyzed, and the surfactant concentration dissolving 1,3-DCB. COC mass balance was checked for the final time by analyzing the COCs in the solvent traps.

The COC volatilization experiments were maintained for 8 h. Then, the airflow was stopped. Table 1 provides a summary of the conditions of the experiments carried out.

In Table 1, all the experiments carried out in the three experiment sets are summarized as follows: from E1 to E17 for surfactant stability (B1), from P1 to P4 for estimation of apparent Henry’s constant (B2), and V1 and V2 for volatilization tests (B3).

### 2.3 Analytical Methods

The pH was analyzed in all experiments (B1, B2, and B3) to verify that pH was >12 using a Metrohm 914 pH/conductometer.

The concentration of COCs in the emulsion in B1 and B3 experimental sets was analyzed by GC. Aqueous samples were diluted 1:10 in methanol and injected in a GC chromatograph (Agilent 8860) using an autosampler (Agilent GC Sampler 120) coupled with an FID and an ECD (GC-FID/ECD). The column was Agilent HP5-MSUI (19091S-433UI, 30 m × 0.25 mm ID × 0.25 μm). 2 μL of samples was injected using helium as carrier gas (flow rate of 2.9 mL·min^–1^). The GC injection port temperature was set at 250 °C, and the GC oven worked at a programed temperature gradient, starting at 80 °C and raising the temperature at a rate of 15 °C·min^–1^ until 180 °C and then keeping it constant for 15 min. Additionally, a split ratio of 10:1 was employed in the analysis.^28^

The COC concentrations in the vapor phase in B2 experiments were measured by HS-GC. GC 20 mL glass vials closed with Teflón caps were filled with 10 mL of alkaline DNAPL-R emulsions. The vials were agitated and heated at the desired temperature for 1 h. After this time, 2.5 mL of the vapor phase was injected into GC using a 10:1 split ratio. The column and the method conditions employed were the same as those described for analyzing dissolved COCs. More details of the method are shown in Table S3.

Surfactant byproducts due to alkaline hydrolysis with the temperature were studied using a Bruker AVANCE 300 MHz spectrometer. 1H NMR spectra of the aqueous samples obtained at the final reaction times in runs E3 and E7 of experimental set B1 and 10 g·L^–1^ of pure samples were recorded. The water content of the samples was previously removed using a rotary evaporator (Büchi Glass Oven B-585) coupled with a vacuum pump (Büchi Vacuum Pump V-300) at 20 °C and 100 mbar for 8 h. The solid residuum was diluted in dimethyl sulfoxide and used as an ISTD analyzed.

## 3 Results and Discussion

### 3.1 Alkaline Hydrolysis of COCs in DNAPL-R

The addition of alkali could be considered a first step to enhancing the volatility of the more highly chlorinated compounds. It was experimentally proved that alkaline pH (>12) promotes the reaction of HCH and PentaCXs to TCBs and HeptaCHs and HexaCXs to TetraCBs in the absence and presence of the surfactant.^29,30^ TCB and TetraCB compounds presented lower risks and lower boiling points than the parent COCs. García-Cervilla et al.^31^ studied the transformation of DNAPL-R at pH 12 in the presence of the surfactant, observing distributing changes since PentaCX, HexaCX, HCH, and HeptaCH isomers were not detected in the aqueous emulsion under alkaline conditions, while the TCB and TetraCB molar percentage was increased under these conditions. Additionally, it was noted that a similar molar total concentration of COCs in solution is obtained independently of the pH employed. The molar distribution of COCs in an emulsion of DNAPL-R at pH 7 and pH 12 is also shown in Table S1.

### 3.2 Surfactant Stability

The influence of the initial surfactant concentration, NaOH concentration, temperature, and presence of COCs on surfactant stability was studied. The experiments are summarized in Table 1.

#### 3.2.1 Effect of the NaOH Concentration (*C*_NaOH_)

The influence of the NaOH concentration was evaluated by varying the concentration between 2 and 4 g·L^–1^ for two different initial surfactant concentrations (5 and 10) g·L^–1^ and two temperatures (20 and 60 °C). Equation 2 calculates the fractional remaining surfactant capacity with time expressed as SCL.
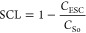
2

**Figure 2:**
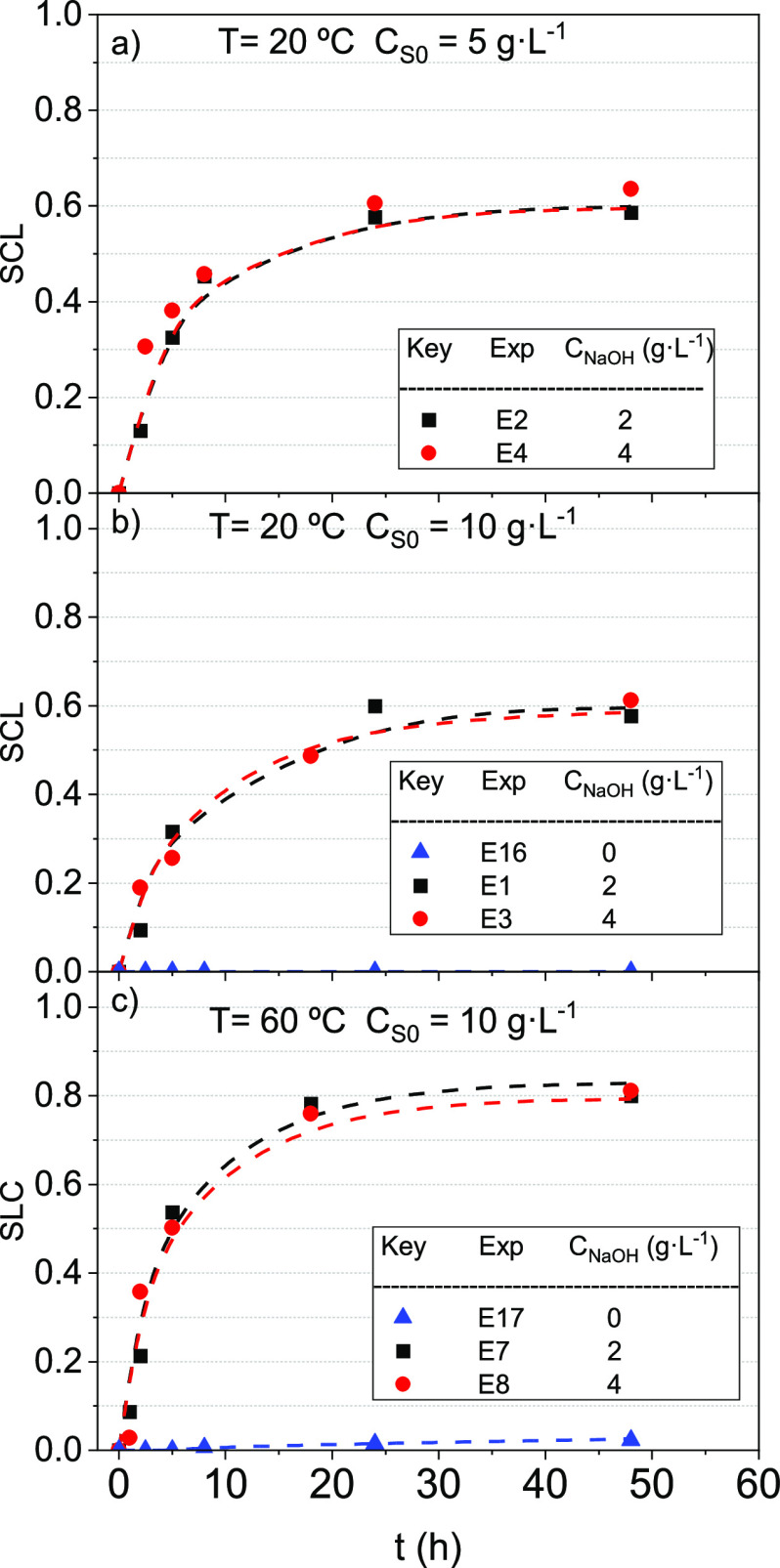
SCL profiles with the time for 2 and 4 g·L^–1^ NaOH at (a) *C*_S0_ = 5 g·L^–1^ and 20 °C, (b) *C*_S0_ = 10 g·L^–1^ and 20 °C, and (c) *C*_S0_ = 10 g·L^–1^ and 60 °C. Symbols indicate experimental results, whereas line values are predicted using the surfactant stability model shown in eq 3.

The SCL profiles with time are shown in Figure 2. As can be seen, in the absence of NaOH in the reaction medium, negligible losses of the surfactant capacity are found under the operation conditions studied. On the contrary, a continuous loss of surfactant capacity (SCL) was observed when alkali was added, with SCL values being lower than 0.05 for both experiments (E16 and E17).

It was previously reported in the literature that OH^–^ anions attack hydrolyzable groups in the surfactant molecule. These transformations produce a consequent loss of the solubilization capacity.^33^ According to the E3 maker, this surfactant is formulated with ethoxylated castor oil, ethoxylated cocamide, ethoxylated fatty acid, and limonene as cosolvents.^34^ These compounds include ester, ether, and double-bond groups, common in polyethoxylated nonionic surfactants such as Triton X, Tween, Brij, Pluronic, and others.^8,35^ Some of these groups are susceptible to hydrolysis under strongly alkaline conditions and temperature.^33^

On the other hand, as shown in Figure 2, no differences were found in the conversion of the surfactant at all the NaOH concentrations tested, regardless of the temperature and the tested initial surfactant concentrations.

#### 3.2.2 Effect of the Initial Surfactant Concentration (*C*_S0_)

**Figure 3:**
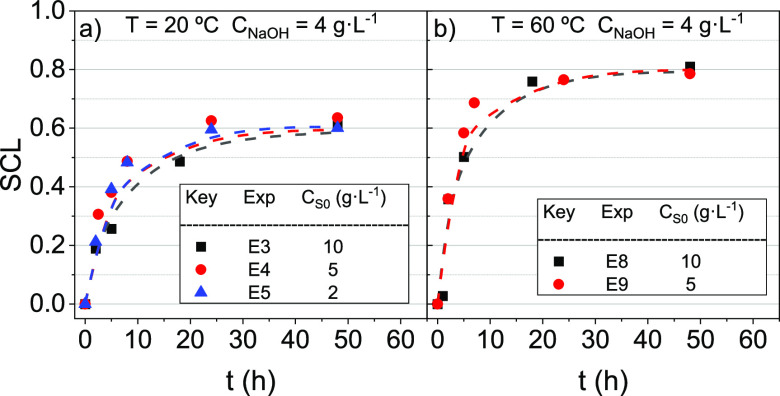
SCL profiles with the time. *C*_NaOH_ was 4 g·L^–1^, and temperature was (a) 20 and (b) 60 °C for an initial concentration of the surfactant of 2, 5, and 10 g·L^–1^. Symbols indicate experimental results, whereas line values are predicted using the surfactant stability model shown in eq 3.

The effect of the initial surfactant concentration on the SCL was investigated at 2, 5, and 10 g·L^–1^ by using two temperatures 20 and 60 °C and keeping constant the NaOH concentration at 4 g·L^–1^. The results obtained are shown in Figure 3. The higher the reaction time, the higher the SCL obtained. Moreover, the SCL was independent of its initial concentration, indicating that the reaction rate of SCL follows a first-order reaction at the surfactant concentration.

As shown in Figure 3, an asymptotic SCL surfactant value was reached in the ranges 0.60–0.64 at 20 °C and 0.79–0.83 at 60 °C. The asymptotic SCL values indicate that final byproducts of surfactant alkaline hydrolysis retain some surfactant capacity to dissolve COCs in the aqueous phase.^36^ In addition, it can be observed that these byproducts were more reactive at higher temperatures, reducing the residual surfactant capacity. The asymptotic value of SCL depends only on temperature and not the surfactant concentration.

#### 3.2.3 Effect of Temperature

**Figure 4:**
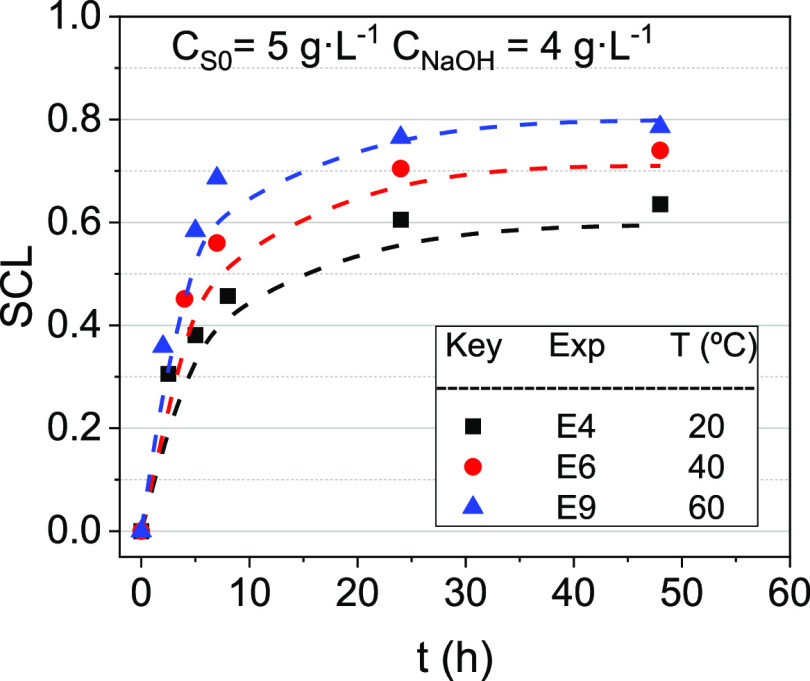
SCL evolution with the time. *C*_S0_ and *C*_NaOH_ were 5 and 4 g·L^–1^, respectively. Temperatures tested were 20, 40, and 60 °C. Symbols indicate experimental results, whereas line values are predicted using the surfactant stability model shown in eq 3.

The temperature effect on SCL was studied in the temperature range 20–60 °C, keeping constant the initial surfactant concentration (5 g·L^–1^) and NaOH concentration (4 g·L^–1^). The evolution of SCL over time is plotted in Figure 4.

As shown in Figure 4, SCL was strongly affected by temperature (20–60 °C). The higher the temperature, the higher the SCL with time. Moreover, as previously commented, the temperature modifies the reactivity of surfactant byproducts, resulting in different asymptotic SCLs (0.64 at 20 °C, 0.74 at 40 °C, and 0.79 at 60 °C).

The byproducts of surfactant hydrolysis were investigated using the nuclear magnetic resonance (NMR) technique. First, the NMR spectrum (shown in Figure S1) of a pure solution of E3 (10 g·L^–1^) was obtained. It has been considered that E3 was composed of castor oil polyethoxylated esters (represented in Figure S2), among others. The NMR spectra of the polyethoxylated group (marked as a red square) and the hydrophobic chain (marked as a green square) were simulated using software included in the SciFinder^n^ application (V11.01 Advanced Chemistry Development, Inc. ACD/LABS). The spectra obtained are summarized in Figures S3 and S4, respectively. Upon comparing the experimental spectra of pure E-Mulse 3 (Figure S1) with the spectra predicted for the polyethoxylated groups and the hydrophobic chain (Figures S3 and S4), the different groups of the surfactant have been identified. The polyethoxylated groups were identified at chemical shifts δ 3.56 and 3.36 ppm. Meanwhile, the chemical shift associated with the unsaturated chain can be located at 5.38 ppm and between 2.10 and 1.31 ppm. The peak located at 5.38 ppm can be attributed to the double-bound on the aliphatic chain (Figure S4).

The surfactant hydrolysis byproducts were investigated using 20 °C (E3) and 60 °C (E7) samples. The NMR spectra of the aqueous solution at the final times in experiments E3 and E7 (set B1 in Table 1) are shown in Figures S5 and S6, respectively. The comparison of results in Figures S5 and S6 with those in Figure S1 reveals that the unsaturated chain disappeared by the effect of NaOH and temperature (Figure S6), confirming the attack of NaOH. On the contrary, the polyethoxylated groups were not attacked by NaOH. Under this experimental evidence, the reaction mechanism was proposed as shown in Figure S7. The surfactant alkaline hydrolysis of ester groups results in the production of an organic sodium salt (C), which maintains some surfactant capacity, and the generation of a byproduct (B) without surfactant capacity (Figure S7). These unsaturated chains were also attacked by NaOH enhanced by temperature reducing the number of intermediates capable of maintaining the surfactant capacity^37^

#### 3.2.4 Effect of COCs in the Emulsion

**Figure 5:**
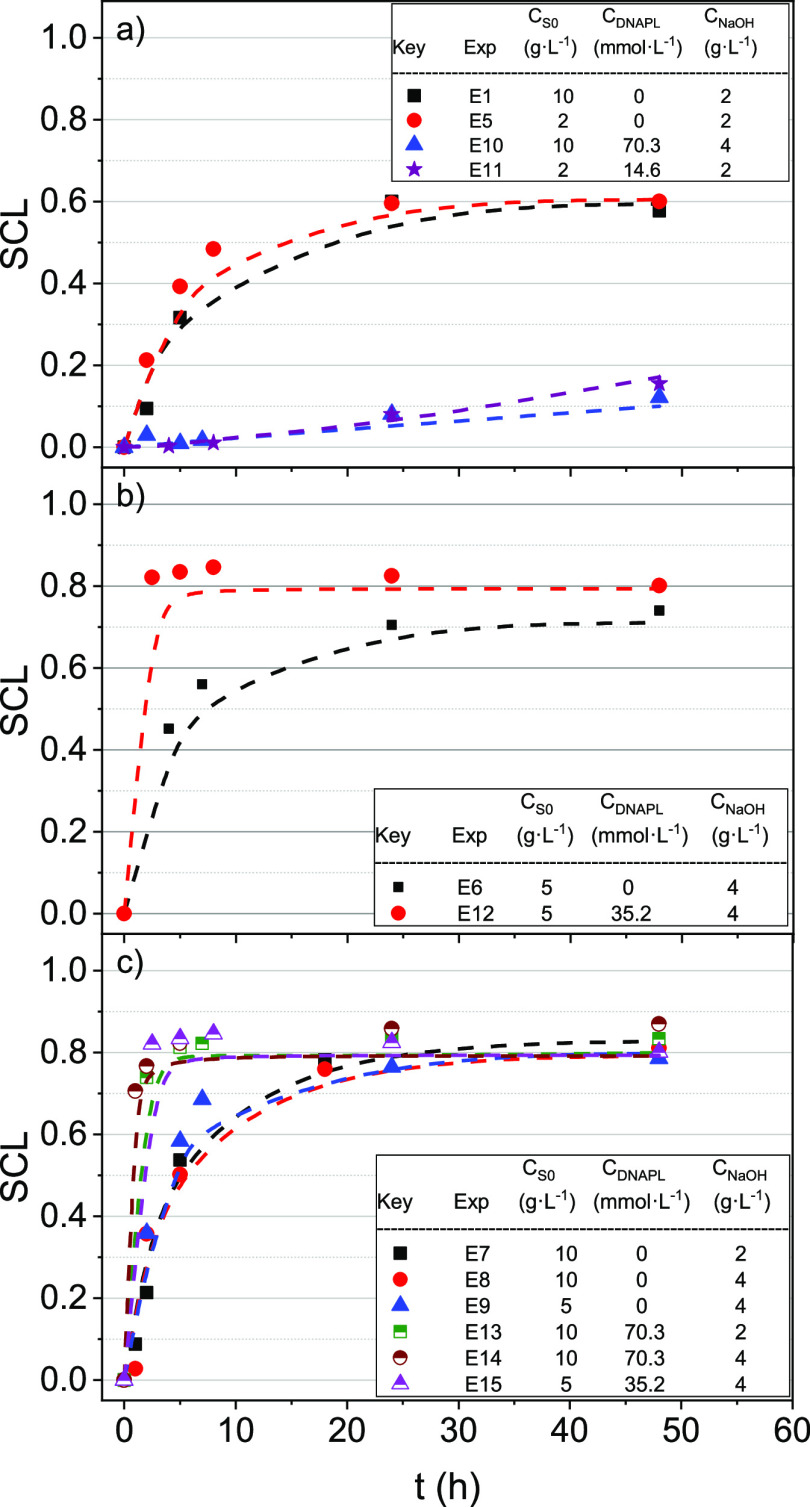
SCL evolution with the time at (a) 20; (b) 40; and (c) 60 °C. The COC concentration was 0 mmol·kg^–1^ and was required for saturation of the initial surfactant solution. Symbols indicate experimental results, whereas line values are predicted using the surfactant stability model shown in eq 3.

The effect of COCs composing DNAPL-S on the SCL was evaluated by adding the amount of DNAPL-S required to reach saturated emulsions (4.33 mmol_COCs_·g_surf_^–1^). The DNAPL-S and initial surfactant concentration ranges used were (14.6, 35.2 and 70.3) mmol·L^–1^ and (2, 5 and 10) g·L^–1^, respectively. Three temperatures were applied (20, 40 and 60 °C), and the corresponding SCL values versus time obtained are shown in Figure 5.

At 20 °C (Figure 5a), it was noticed that COCs in the emulsion inhibited the SCL. At 48 h, an SCL of about 15% is obtained in experiments E10 and E11, whereas in experiments E1 and E5 (without COCs in the solution), SCL reaches 60% at the same time. However, as the temperature increases, this inhibition disappears, as shown in Figure 5b,c.

#### 3.2.5 Modeling the SCL Rate

The effect of studied variables on the SCL of surfactant E3 has been taken into account by a kinetic model predicting the SCL rate. With the experimental results, the following assumptions have been made:

- The partial order of NaOH in the SCL reaction rate is zero.
- The SCL follows a first-order reaction on the surfactant.
- The SCL asymptotic value depends on the temperature.
- The COCs in the emulsion inhibit the SCL, but this effect changes as the temperature increases. The proposed kinetic model can be used in the presence and absence of COCs in the emulsion. The different influence of COCs at high or low temperature has been taken into account using *k*_1_ and *k*_2_ in eq 3.

With these assumptions, the proposed kinetic model of the SCL rate is shown in eq 3.
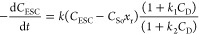
3where *C*_ESC_ and *C*_S0_ are the surfactant equivalent concentration (g·L^–1^) at a time *t* and the surfactant concentration at zero time, respectively; *C*_D_ is the COC concentration (mmol·L^–1^) at a time; *C*_NaOH_ is the NaOH concentration (g·L^–1^); and *k* is the reaction rate constant (h^–1^). *K*_1_ and *k*_2_ in (L·mmol^–1^) are constants that take into account the effect of COCs on SCL with the temperature. *k*, *k*_1_, and *k*_2_ follow the Arrhenius law,^38^ expressed in eqs 4–6, respectively; *x*_r_ is the residual surfactant with temperature, being a function of the temperature as proposed in eq 7.
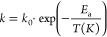
4
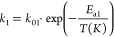
5
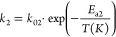
6
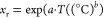
7

The experiments in this section using emulsions with COCs were carried out by saturating the surfactant emulsion with DNAPL. The amount of solubilized COCs in a saturated emulsion is linear with the surfactant concentration at alkaline pH.^30^ Therefore, the decrease in the surfactant concentration with time produces a decrease in the COCs in the emulsion *C*_D_ when saturated emulsions are used at zero time. The remaining surfactant concentration with time *C*_D_ can be calculated using eq 8.
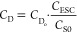
8

Data from experiments in set B1 were fitted to the model in eqs. 3–7. The problem to be solved is composed of a mixed set of differential and algebraic equations. It was implemented in ModelBuilder 7.1.0 provided in the gPROMS suit, and the algorithm DASOLV was used to simulate the reaction system. DASOLV is based on a variable time step, variable order, and backward differentiation formulae.^39^ The estimated parameters calculated for the SCL kinetic model by minimizing the sum of quadratic squares (eq 9) are shown in

Table 2, with the confidence interval (CI) (95%) of the parameters.
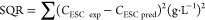
9

**Table 2:** Parameters Estimated for the SCL Rate^a^

*E*_a_ (K)	724.1 ± 31.5
*E*_a1_ (K)	–2575 ± 112
*E*_a2_ (K)	–20835 ± 918
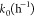	1.97 ± 0.06
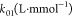	0.27 ± 0.03
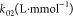	1.4 × 10–^5^ ± 0.2 × 10–^5^
*A*	–0.57 ± 0.02
*b*	–1.68 × 10–^2^ ± 7.36 × 10–^4^
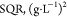	3.2

a 95% CI.

### 3.3 Surface Responses for the Apparent Henry’s Constant

The volatilization of COCs from the emulsion formed by DNAPL and E3 was studied in experiments summarized in Table 1. The vapor–liquid equilibrium (VLE) of component *j* can be described by Henry’s law^22^ assuming that the vapor phase is an ideal gas phase, and the fugacity can be considered close to unity.^40^ The Henry’s law in eq 10 was formulated using an apparent Henry’s constant in surfactant solutions.^22^ This constant considers the COCs to be partitioned into vapor, extramicellar (aqueous), and micellar phases.^23^
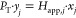
10

where *P*_T_ is the total pressure in the vial (bar) at the temperature *T*; *y*_*j*_ is the molar fraction of COC *j* in the vapor phase; *H*_app,*j*_ is the apparent Henry’s constant of compound *j*; and *x*_*j*_ is the molar fraction of compound *j* in the liquid phase. The molar fraction in the liquid phase was calculated by mass balance as the difference between the amount of compound *j* in the vapor phase and the initial amount prepared in the sample.

In eq 10, the total pressure in the vial (bar) is calculated assuming that under the conditions tested, water and air are the main compounds in the gas phase in the vial, according to eq 11.
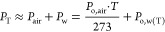
11

where *P*_0 air_ is the initial pressure of the vial at 20 °C (water in the phase can be neglected at this temperature) and *P*_w_ is the water pressure in the vial gas phase at corresponding *T* (equal to water vapor pressure at *T*, assuming that the molar fraction of water in the liquid phase is almost the unity).

The presence of the surfactant (concentration and type) in the aqueous phase can modify Henry’s constant of the chlorinated compound *j*.^24^ Also, the complex mixture of DNAPL can affect this constant.

Experimental values of *H*_app,*j*_ for each compound *j* at different temperatures and surfactant and COC concentrations in the liquid phase were determined according to eq 12, after measuring the gas phase composition of the vial by GC, as explained in the Analytical Methods section.
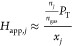
12where *n*_*j*_ is the moles of the *j* compound in the vial gas phase and *n*_gas_ is the sum of moles of all compounds (including organic, air, and water) in the vial gas phase.

The experimental values of  obtained at different temperatures, surfactant concentrations, and COC concentrations in the aqueous phase are shown as red points in Figure S8. The different red points, for the same values of the temperature and surfactant concentration, refer to the different COC concentrations used at those values of *T* and *C*_S0_ (experimental conditions are detailed in Table 1). The influence of the COC concentration in emulsion on ln(*H*_app,*j*_*)* can be neglected if the surfactant concentration and temperature keep constant, as shown in Figure S8. On the contrary, a positive effect of temperature on ln(*H*_*app*,*j*_) was found. As expected, organic compounds in the emulsion have a significant tendency to pass to the vapor phase as the temperature increases. On the contrary, the higher the surfactant concentration, the lower the ln(*H*_app,*j*_) value of the *j* compound. The increase of the surfactant concentration results in a higher concentration of micelles,^12^ inhibiting the volatilization of chlorinated compounds from the emulsion, which agrees with the conclusion reported in the literature.^24^

The interaction between the surfactant concentration and temperature at *H*_app,*j*_ values has been modeled using the response surface methodology (RSM). Experimental values of *H*_app,*j*_ shown in Figure S8 have been fitted to eq 13.
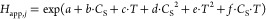
13where *T* is the temperature (°C) and *C*_S_ is the surfactant concentration (g·L^–1^) when VLE is reached. Equilibrium was achieved in 1 h, and corresponding *C*_S_ was calculated using the kinetic model proposed in Section 3.2.5. Modeling of the SCL rate has bee summarized in Table S4.

The estimated values of parameters *a*–*f* in eq 13 and the statistical parameters obtained from the variance analysis [coefficient of variation (*R*^2^), Fischer’s test value (*F*-value), and probability (*p*-value)] are summarized in Table 3. As can be seen, the value of *R*^2^ is close to 1 for all the compounds present in DNAPL-R, indicating a good agreement between experimental and predicted values. Additionally, the *F*-values are large (≫1), and the *p*-values are small enough (<0.05) for all the chlorinated compounds studied. Therefore, the RSM model applied and the parameters obtained allow us to estimate accurately the *H*_app,*j*_ values of each *j* compound as a function of the surfactant concentration and temperature, with the negligible effect of the COC concentration in the emulsion.

**Table 3:** Parameters Obtained from the Fitting of *H*_app,*j*_ to eq 13^a^

	*a*	*b*	*c*	*d*	*E*	*f*	*R*^2^	*F*-value	*p*-value
CB	2.54	–0.24	0.08	8.52 × 10^–^^3^	–4.39 × 10^–^^4^	6.90 × 10^–^^5^	0.99	349	4.42 × 10^–^^19^
1,4-DCB	0.26	–0.34	0.11	1.35 × 10^–^^2^	–7.73 × 10^–^^4^	–3.68 × 10^–^^4^	0.97	173	6.77 × 10^–^^17^
1,2-DCB	0.16	–0.37	0.13	1.44 × 10^–^^2^	–9.25 × 10^–^^4^	–4.64 × 10^–^^5^	0.97	136	1.09 × 10^–^^17^
1,2,4-TCB	–3.94	–0.37	0.26	1.84 × 10^–^^2^	–2.03 × 10^–^^3^	–2.36 × 10^–^^3^	0.97	408	1.08 × 10^–^^15^
1,2,3-TCB	–4.69	–0.37	0.26	1.69 × 10^–^^2^	–1.98 × 10^–^^3^	–2.08 × 10^–^^3^	0.98	171	4.61 × 10^–^^19^
a-TetraCB	–5.28	–0.60	0.27	2.63 × 10^–^^2^	–2.19 × 10^–^^3^	1.04 × 10^–^^4^	0.98	320	1.82 × 10^–^^20^
b-TetraCB	–6.05	–0.57	0.28	2.60 × 10^–^^2^	–2.17 × 10^–^^3^	–4.11 × 10^–^^4^	0.98	325	1.53 × 10^–^^20^

a The statistical parameters were obtained from variance analysis. The coefficient of variation (*R*^2^), Fischer’s test value (*F*-value), and probability (*p*-value) are also shown.

### 3.4 Volatilization of COCs from Alkaline Emulsions

Emulsion of DNAPL obtained in SEAR treatment must be treated to eliminate the organic compounds. In the case of DNAPL from lindane liquid wastes, a significant fraction of COCs in emulsion correspond to low volatile HCHs and HeptaCHs. Therefore, as cited before, the previous alkalinization transforms these compounds into more volatile TCBs and TetraCBs. Volatilization of COCs in the alkaline solution can be modeled by considering those values that influence the volatility of the COCs. These variables are temperature and surfactant concentrations in the aqueous phase. In addition, the surfactant concentration in the emulsion can change with time according to the SCL rate equation described in Section 3.2.5. Modeling SCL rate.

The molar balance of COC *j* in the emulsion in the batch experiment schematized in Figure 1 can be calculated using eq 14.
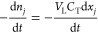
14where *n*_*j*_ is the moles of *j* in the emulsion; *V*_L_ is the volume of the aqueous emulsion (L); *C*_T_ is the total molar concentration of the emulsion (approximately corresponding to water: 55 mol·L^–1^); and *x*_*j*_ is the molar fraction of the compound *j* in the liquid phase.

The gas flow rate leaving the bottle (Figure 1) is assumed to be in equilibrium with the emulsion by applying the Raoult law. The molar fraction of the *j* compound in the gas phase is calculated with eq 15.
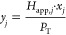
15

The molar flow rate of the *j* compound disappearing from the emulsion is the same as the molar flow of this *j* compound that leaves the bottle in the gas phase (both phases in equilibrium), as described in eq 16.
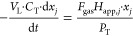
16where *F*_gas_ is the gas molar flow rate (mol·h^–1^) fed to the system.

The molar fraction of *j* in the emulsion with time can be predicted by integrating eq 16 as shown in eq 17.
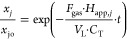
17

The ratio *x*_*j*_/*x*_*j*o_ also corresponds to the concentration ratio of the *j* compound in the emulsion (eq 18)
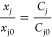
18where *C*_*j*_ is the concentration of *j* in the aqueous emulsion a time *t* and *C*_*j*0_ is the concentration of *j* in the aqueous emulsion before the gas is fed (zero time). The concentration of the sum of COCs remaining in the emulsion can be estimated from eq 19
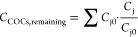
19

The value of *H*_app,*j*_ at each time is obtained by eq 13, with the surfactant concentration predicted by eq 3 at the time considered.

**Figure 6:**
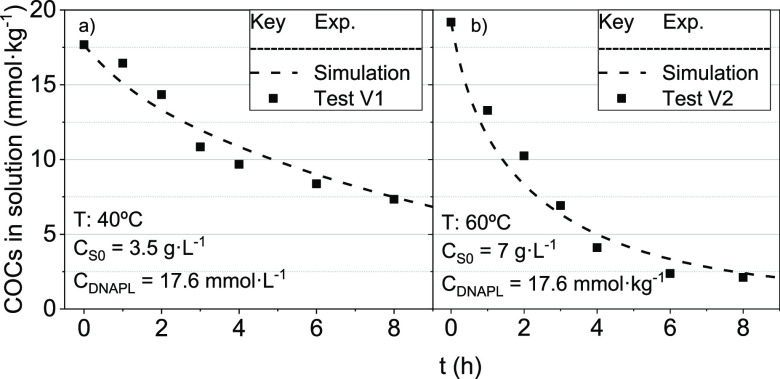
Volatilization of COCs in the emulsion. (a) *T* = 40 °C; *C*_S0_ = 3.5 g·L^–1^ and (b) *T* = 60 °C; *C*_S0_ = 7 g·L^–1^. Conditions *C*_DNAPL-S_ = 17.6 mmol·L^–1^; *V*_aq_ = 0.25 L; air flow 3 L·h^–1^; and *C*_NaOH_ 4 g·L^–1^ NaOH. Symbols indicate experimental results, whereas line values are predicted using eq 17.

The consistency of the SCL kinetic model and *H*_app,*j*_ obtained in the surfactant presence were validated by comparing the experimental and predicted values of COCs in the emulsion obtained in runs shown in Table 1 (set B3). Two different temperatures were employed (40 and 60 °C) as two different initial surfactant concentrations (3.5 and 7 g·L^–1^) have been used. The gas flow rate employed was 3 L·h^–1^, the emulsion volume was 0.25 L, and the initial COC concentration in the emulsion was 17.6 mmol·kg^–1^. DNAPL-S was used, with a similar composition to DNAPL-R after alkalinization. The air was flowing during 8 h. Experimental values with time of COCs in emulsion (as symbols) and those predicted with eq 17 (as lines) are shown in Figure 6. As can be seen, a good agreement is obtained between experimental and predicted COCs.

**Figure 7:**
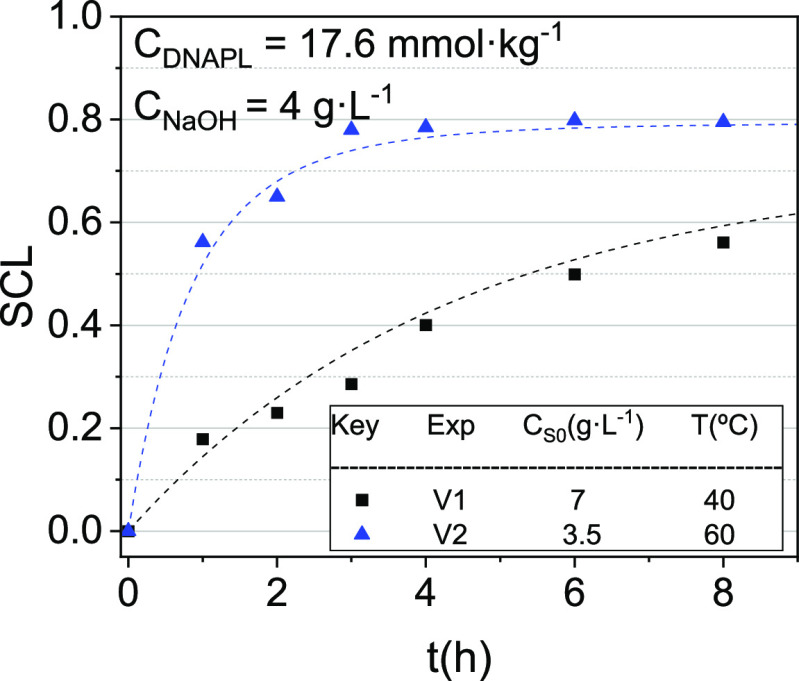
Evolution of SCL with the time for the volatilization experiments. Symbols indicate experimental results, whereas line values are predicted using eq 17.

The SCL during volatilization has also been measured and estimated for runs shown in Table 1. Experimental and predicted values are shown in Figure 7. The excellent agreement found between the observed and predicted values of SCL inferred that the significant losses of surfactant capacity were related to the reaction between the surfactant and NaOH. The losses of the surfactant by foaming are negligible. As observed with the volatilization experiments, the SCL reached 0.8 at 60 °C and 0.62 at 40 °C during 8 h of treatment. Therefore, volatilization can be employed not only to remove COCs from emulsions but also to break them and thus facilitate their further disposal. The reduction of surfactant and pollutant contents in the processed stream permits the treatment of the water stream obtained after the air stripping step under alkali conditions, avoiding other expensive technologies such as incineration in special facilities.^41^

## 4 Conclusions

In this work, a complex mixture of chlorinated organic contaminants in a surfactant emulsion simulating a SEAR stream was successfully treated by air stripping. The emulsion was alkalinized to transform the original pollutants (PentaCX, HCH, and HeptaCH) to more volatile compounds (triCB and tetraCB).

The air-stripping treatment design required studying the volatilization of COCs and the SCL. Both approaches were affected by temperature and NaOH, surfactant, and COC concentrations. It was found that temperature and alkali produced the SCL with time. Under alkaline conditions, the OH^–^ anions attack hydrolyzable groups in the surfactant molecule, resulting in the loss of unsaturated chains. The surfactant byproducts of alkaline hydrolysis keep some residual surfactant capacity (about 0.36 to 0.21 of the initial value). The variables’ effect in the SCL was used to develop a kinetic model that can adequately explain the experimental findings.

In addition, it was observed that the surfactant presence drastically reduced the volatilization of those COCs, and their volatilization increased with temperature, while the COC concentration in the emulsion did not affect the volatilization of the COCs. The *H*_app,*j*_ values obtained have been adequately correlated with the variables studied using surface response methodology.

The volatilization of COCs in the alkaline emulsion by air stripping was experimentally measured and predicted. The air stripping under alkali conditions successfully reduced the initial concentration of COCs by more than 90% after 8 h at 60 °C. In addition, SCL during air stripping was higher than 80% at 60 °C, making the emulsion disposal more straightforward. The simulated values of COCs in emulsion with time using the kinetic model of surfactant stability and the *P*_*v*_^o^γ correlations agree well with the experimental results, validating the model.

The volatilization of COCs by air stripping was successfully applied to move and concentrate these compounds to the vapor phase. This stream can be treated by coupling different technologies, such as the adsorption in AC, whose efficiency is improved when the surfactant is removed from the fed stream.

## Supporting Information Available

Additional details about the composition of DNAPL-R used and of synthetic DNAPL; additional details regarding the analysis method; NMR spectra of E3; chemical structure of a polyethoxylated ester which can be found in the surfactant employed; NMR spectrums predicted for the polyethoxylated group and aliphatic chain; NMR spectra for different experiments carried out in the article; mechanism of surfactant alkaline hydrolysis proposed; and values of  for the different experiments carried out and the response surfaces for the different compounds (PDF)

## Abbreviations.

AC: activated carbon
BDF: backward differentiation formulae
CB: chlorobenzene
CI: 95% confidence interval
CMC: critical micelle concentration
COCs: chlorinated organic compounds
DCB: dichlorobenzene
DCE: dichloroethene
DMSO: dimethyl sulfoxide
DNAPL: dense non-aqueous phase liquid
DNAPL-R: real dense non-aqueous phase liquid
DNAPL-S: synthetic dense non-aqueous phase liquid
E3: E-Mulse 3
ECD: electron capture detector
ESC: equivalent surfactant concentration
FID: flame ionization detector
GC: gas chromatography
HCHs: hexachlorocyclohexane isomers
HeptaCHs: heptachlorocyclohexane isomers
HexaCXs: hexachlorocyclohexane isomers
HS-GC: HeadSpace gas chromatography
ISTDs =: internal standards
MeOH: methanol
MSR: molar solubilization ratio
NAPLs: non-aqueous phase liquids
NMR: nuclear magnetic resonance
PAHs: polycyclic aromatic hydrocarbons
PCE: tetrachloroethene
PentaCXs: pentylcyclohexanes
RSM: response surface methodology
SCL: surfactant capacity loss
SDS: sodium dodecyl sulfate
SDBS: sodium dodecyl benzene sulfonate
SEAR: surfactant enhanced aquifer remediation
SQR: sum of quadratic squares
TCB: trichlorobenzene isomers
TCE: trichloroethene
TetraCB: tetrachlorobenzene isomers
TPH: total petroleum hydrocarbons
VLE: vapor–liquid equilibrium

## Symbols

*a*: fitting parameter
*b*: fitting parameter
*c*: fitting parameter
*C*_*j*_: concentration of compound *j* in mmol·L^–1^*(j* = COCs, DNAPL) or g·L^–1^ [*j* = surfactant (S), ESC, NaOH]
CTtotal molar concentration of the emulsion (approximately corresponding to water: total molar concentration of the emulsion (approximately corresponding to water55 mol·L^–1^)
*d*: fitting parameter
*e*: fitting parameter
Ea: activation energy in K.
*f*: fitting parameter
*F*_gas_: gas molar flow rate in mol h^–1^
*H*_app,*j*_: apparent Henry’s constant of compound *j* in the emulsion in bar.
*k*: reaction rate constant in h^–1^.
*k*_0_: preexponential factor in L·mmol^–1^
*k*_*i*_: constants that take into account the effect of COCs on SCL in L·mmol^–1^.
*n*_*j*_: mole of compound *j* in mol
*P*: pressure in bar
*P*_v*j*_: saturation vapor pressure of compound *j* in bar
*T*: temperature in °C
*t*: time in h
*V*L: volume of the aqueous emulsion in *L*
*x*_*j*_: molar fraction of compound *j* in the liquid phase
*x*_r_: residual surfactant with temperature
*y*_*j*_: molar fraction of compound *j* in the vapor phase

## Greek Letters

γ_*j*_: activity coefficient of the chlorinated compound *j*

## Subscripts

0: initial
air: air
D: COC concentration
exp: experimental value
gas: vapor phase
pred: predicted value
*s*: surfactant
*T*: temperature
*w*: water
