# Supplementary material for: Treatment of a Complex Emulsion of a Surfactant with Chlorinated Organic Compounds from Lindane Wastes under Alkaline Conditions by Air Stripping
Source: Ind Eng Chem Res. 2023 Feb 7;62(7):3282–93. doi: 10.1021/acs.iecr.2c03722 (PMC9951212; doi:10.1021/acs.iecr.2c03722)
Supplement: Supplementary file 1 — ie2c03722_si_001.pdf [file ie2c03722_si_001.pdf]

Treatment of a complex emulsion of surfactant with chlorinated organic compounds from lindane wastes at alkaline conditions by air stripping.

Patricia Sáez[a], Raúl García-Cervilla[a], Aurora Santos[a], Arturo Romero[a], David Lorenzo[a]*

*dlorenzo@quim.ucm.es

[a]Chemical Engineering and Materials Department. Complutense University of Madrid. Spain.

Supporting Information

Table S1. DNAPL PS14D composition in $mol{\cdot}mol^{-1}$.

**Acronym**	**Name**	**CAS**	**MW**	$x_i\,pH\,7$	$x_i\,pH > 12$
CB	Chlorobenzene	108-90-7	113	0.288	0.187
1,3-DCB	1,3-Dichlorobenzene	541-73-1	147	0.006	0.009
1,4-DCB	1,4-Dichlorobenzene	106-46-7	147	0.080	0.080
1,2-DCB	1,2-Dichlorobenzene	95-50-1	147	0.075	0.065
1,3,5-TCB	1,3,5-Trichlorobenzene	108-70-3	181	0.001	0.007
1,2,4-TCB	1,2,4-Trichlorobenzene	120-82-1	181	0.123	0.349
1,2,3-TCB	1,2,3-Trichlorobenzene	87-61-6	181	0.017	0.054
TetraCB-a	1,2,4,5-Tetrachlorobenzene<br>1,2,3,5-Tetrachlorobenzene	95-94-3<br>634-90-2	216	0.024	0.096
TetraCB-b	1,2,3,4-Tetrachlorobenzene	634-66-2	216	0.041	0.154
γ-PentaCX	γ-Pentachlorocyclohexene	342631-17-8	254	0.020	-
PentaCB	1,2,3,4,5-Pentachlorobenzene	608-93-5	250	0.002	-
δ-PentaCX	δ-Pentachlorocyclohexene	643-15-2	254	0.031	-
θ-PentaCX	θ-Pentachlorocyclohexene	319-94-8	254	0.003	-
HexaCX-a	Hexachlorocyclohexene	1890-41-1	289	0.004	-
β-PentaCX	β-Pentachlorocyclohexene	319-94-8	254	0.003	-
η-PentaCX	η-Pentachlorocyclohexene	54083-24-8	254	0.003	-
HexaCX-b	Hexachlorocyclohexene	1890-41-1	289	0.002	-
HexaCX-c	Hexachlorocyclohexene	1890-41-1	289	0.004	-
α-HCH	α-Hexachlorocyclohexane	319-84-6	291	0.029	-
HexaCX-d	Hexachlorocyclohexene	1890-41-1	291	0.001	-
β-HCH	β-Hexachlorocyclohexane	319-85-7	291	0.001	-
γ-HCH	γ-Hexachlorocyclohexane	58-89-9	291	0.084	-
HeptaCH-1	Heptachlorocyclohexane	707-55-1	325	0.037	-
δ-HCH	δ-Hexachlorocyclohexane	319-86-8	291	0.085	-
ε-HCH	ε-Hexachlorocyclohexane	6108-10-7	291	0.013	-
HeptaCH-2	Heptachlorocyclohexane	707-55-1	325	0.017	-
HeptaCH-3	Heptachlorocyclohexane	707-55-2	325	0.008	-

Table S2. Synthetic DNAPL composition in $mol \cdot mol^{-1}$.

**Acronym**	**Name**	**CAS**	**MW**	**DNAPL-R** x_i	**DNAPL-S** x_i
CB	Chlorobenzene	108-90-7	113	0.187	0.274
1,2-DCB	1,2-Dichlorobenzene	95-50-1	147	0.154[a]	0.142
1,2,4-TCB	1,2,4-Trichlorobenzene	120-82-1	181	0.349	0.335
1,2,3 TCB	1,2,3-Trichlorobenzene	87-61-6	181	0.061[b]	0.051
TetraCB-a	1,2,4,5-Tetrachlorobenzene<br>1,2,3,5-Tetrachlorobenzene	95-94-3<br>634-90-2	216	0.096	0.058
TetraCB-b	1,2,3,4-Tetrachlorobenzene	634-66-2	216	0.154	0.14

a. Dichlorobenzenes isomers in DNAPL-R were lumped as 1,2-DCB

b. The 1,2,3-DCB and 1,3,5-DCB in DNAPL-R were lumped as 1,2,3 DCB

Table S3. HS-GC conditions.

Parameters	Conditions
Headspace	
Incubation Temperature (ºC)	30, 40, 60
Incubation time (min)	60
Syringe Temperature (ºC)	90
Agitator Speed (r.p.m.)	500
Gas Cromatograph	
Carrier gas	Helium
Carrier gas pressure (kPa)	22.7
Injector Temperature (ºC)	180
Injection Volume (mL)	2.5
Split Ratio	10:1
Split Flow ($mL\cdot min^{-1}$)	50
Temperature Program	Initial Temperature 80 ºC<br>Ramping to 80 ºC at 180ºC hold for 9 min
Detectors FID	Heater: 300ºC<br>Air Flow: 300 mL/min<br>H_2 Fuel Flow: 40 mL/min<br>Makeup Flow N_2: 40 mL/min
Detectors ECD	Heater: 210ºC<br>Makeup Flow N_2: 40 mL/min

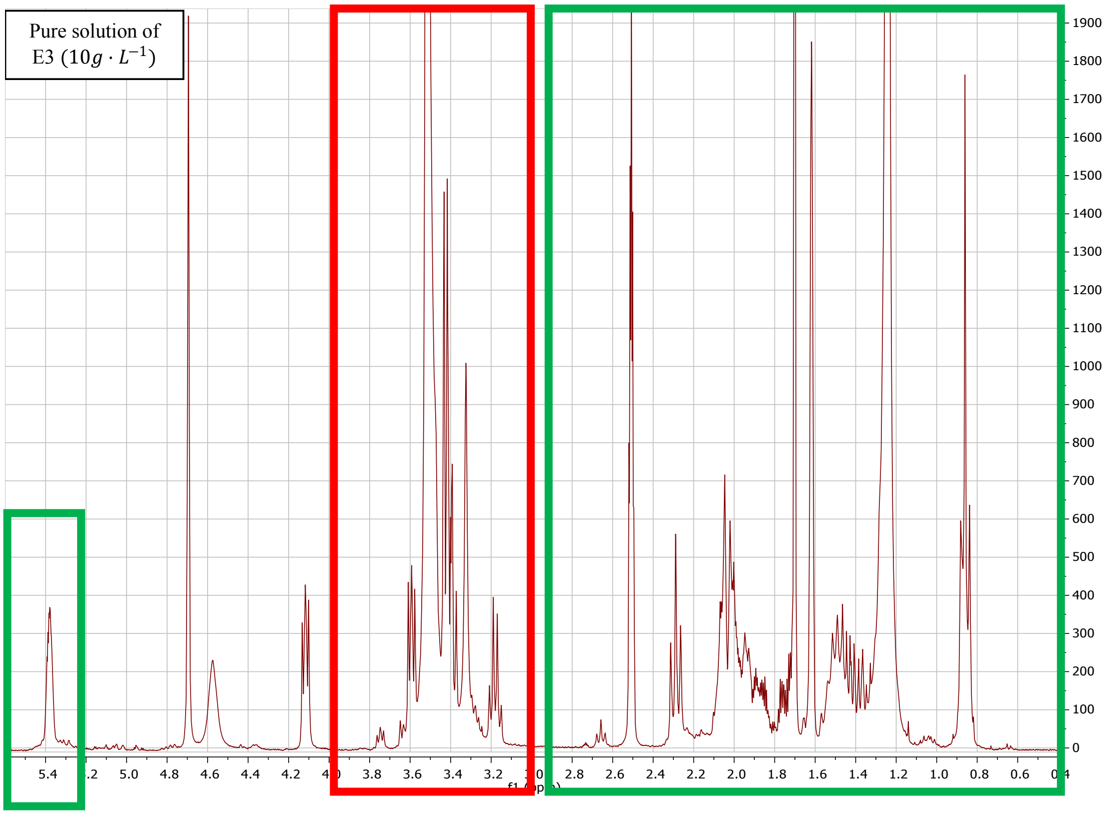

Figure S1. NMR spectra of pure solution of E3 at 10 $g{\cdot}L^{-1}$. The red square depicts the polyethoxylated group whose NMR spectrum was predicted as is shown in Figure S3. The green square depicts the aliphatic chain used to predict the NMR spectra in Figure S4.

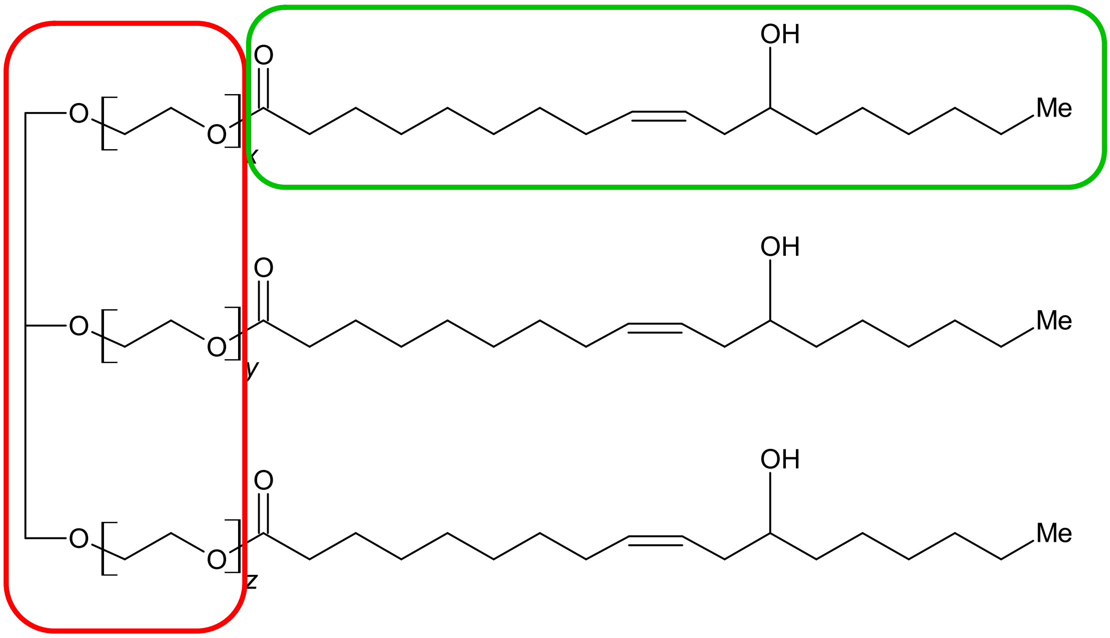

Figure S2. Chemical structured of a polyethoxylated ester of castor oil, which can be found in the formulation of E3. The red square depicts the polyethoxylated group whose NMR spectrum was predicted as is shown in Figure S3. The green square depicts the aliphatic chain used to predict the NMR spectra in Figure S4.

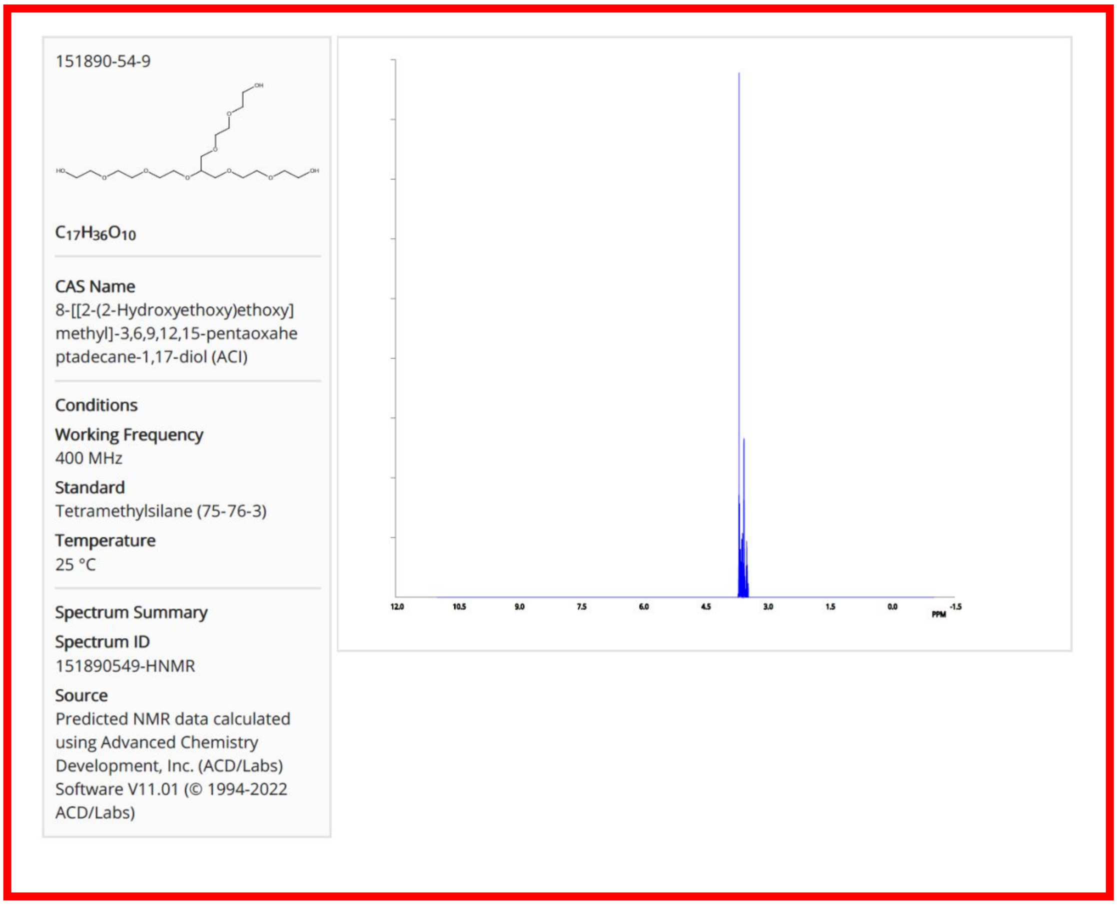

Figure S3. NMR spectrum predicted for the polyethoxylated group marked as red square in Figures S1 and S2. Spectrum obtained from software included in the SciFinder^n application (V11.01 Advanced Chemistry Development, Inc. ACD/LABS).

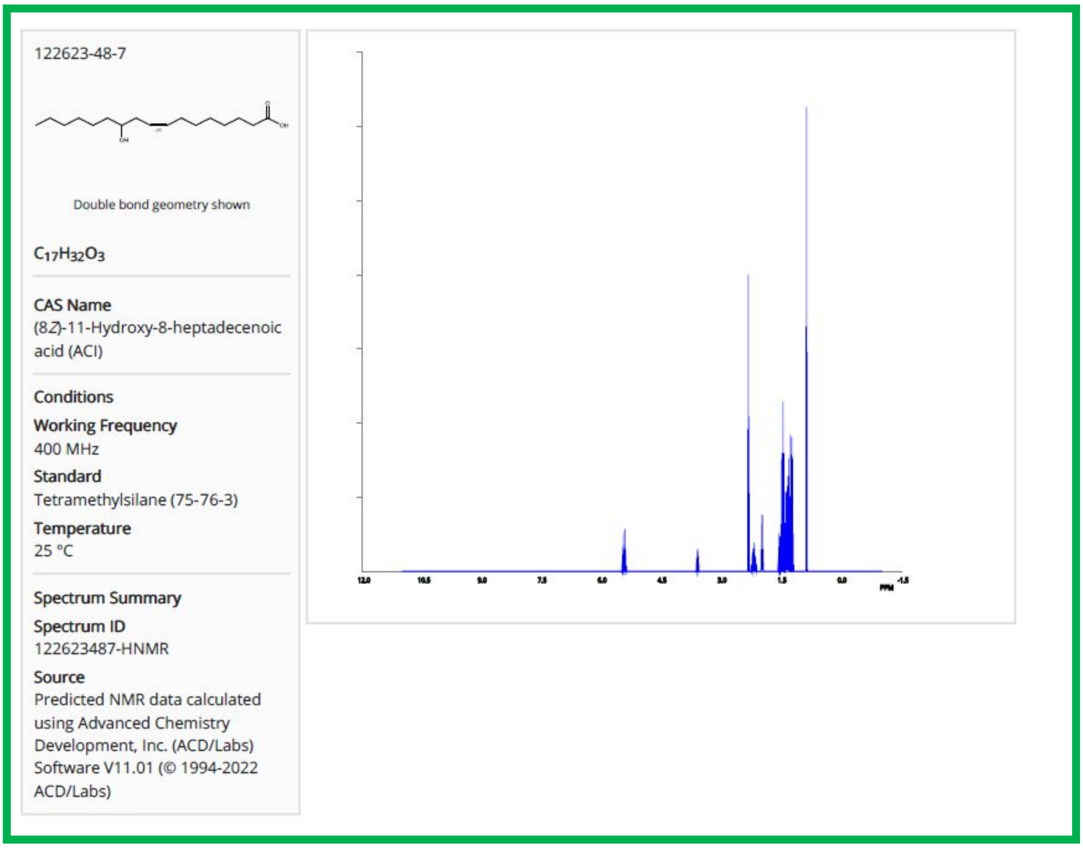

Figure S4.. NMR spectrum predicted for the aliphatic chain marked as green square in Figures S1 and S2. Spectrum obtained from software included in the SciFinder^{n} application (V11.01 Advanced Chemistry Development, Inc. ACD/LABS).

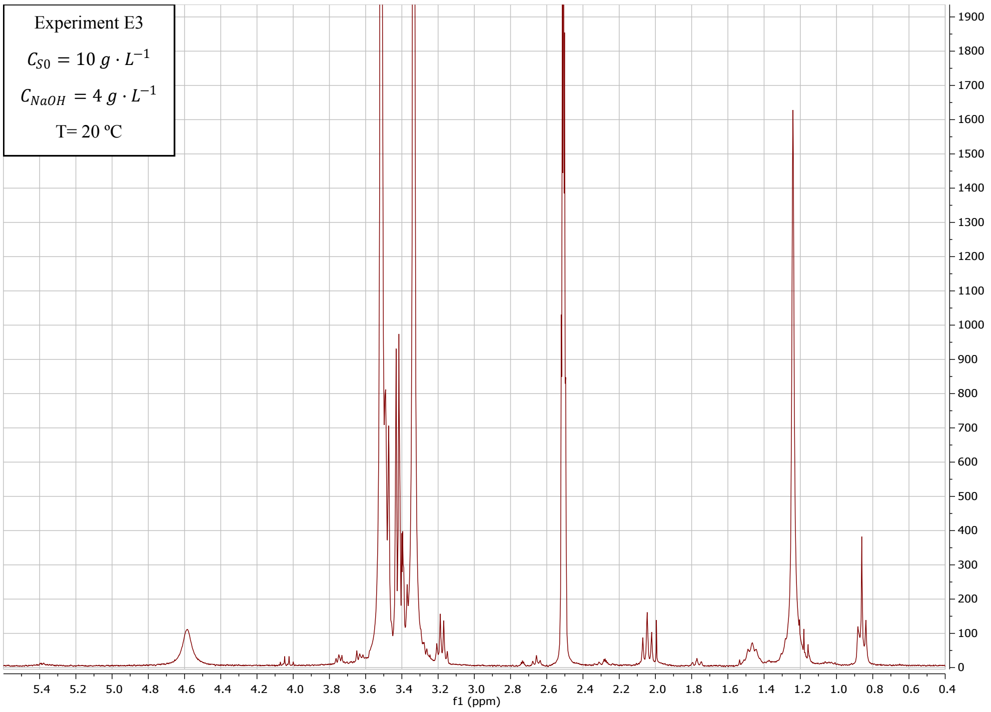

Figure S5. NMR spectra of experiment E3 (Conditions: $C_{S0} = 10\ g{\cdot}L^{-1}$;$C_{NaOH} = 4g{\cdot}L^{-1}$;$T = 20\ °C$).

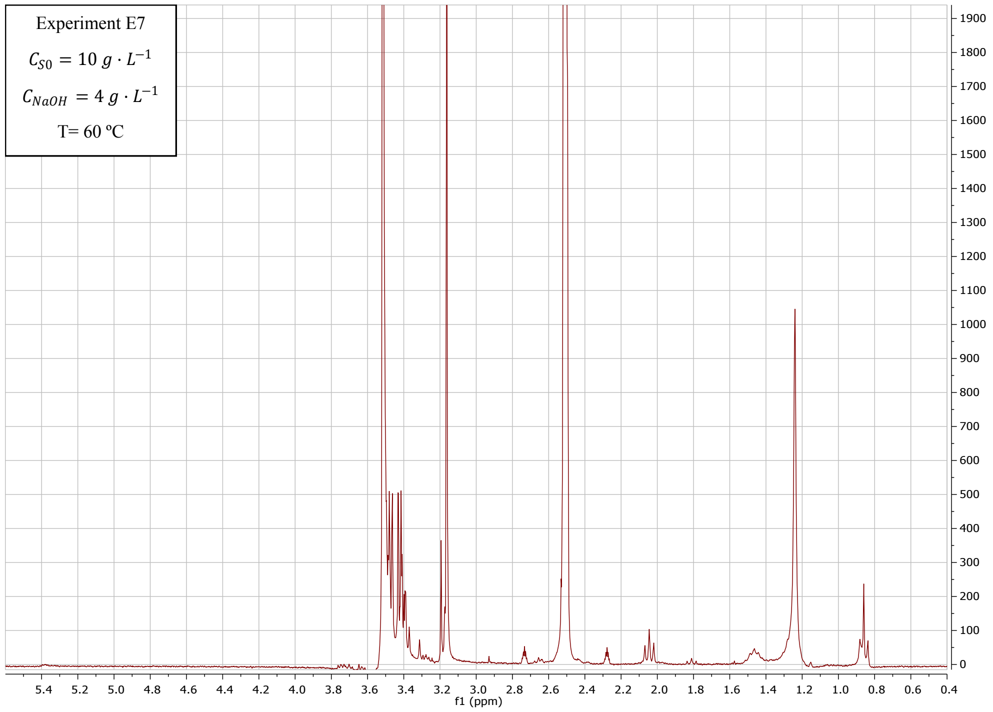

Figure S6. NMR spectra of experiment E7 (Conditions: $C_{S0} = 10\ g{\cdot}L^{-1}$;$C_{NaOH} = 4g{\cdot}L^{-1}$;$T = 60\ °C$).

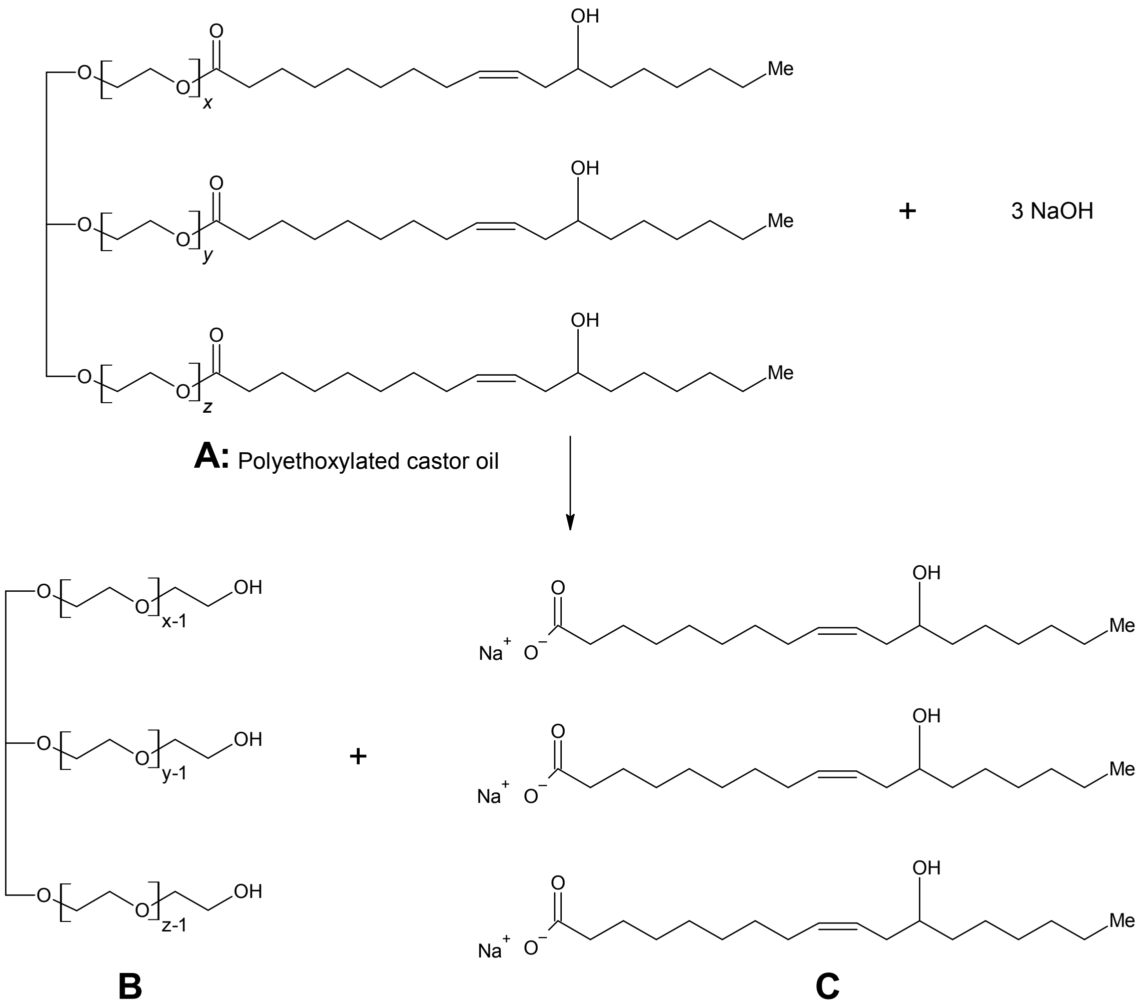

Figure S7. Mechanism of surfactant alkaline hydrolysis.

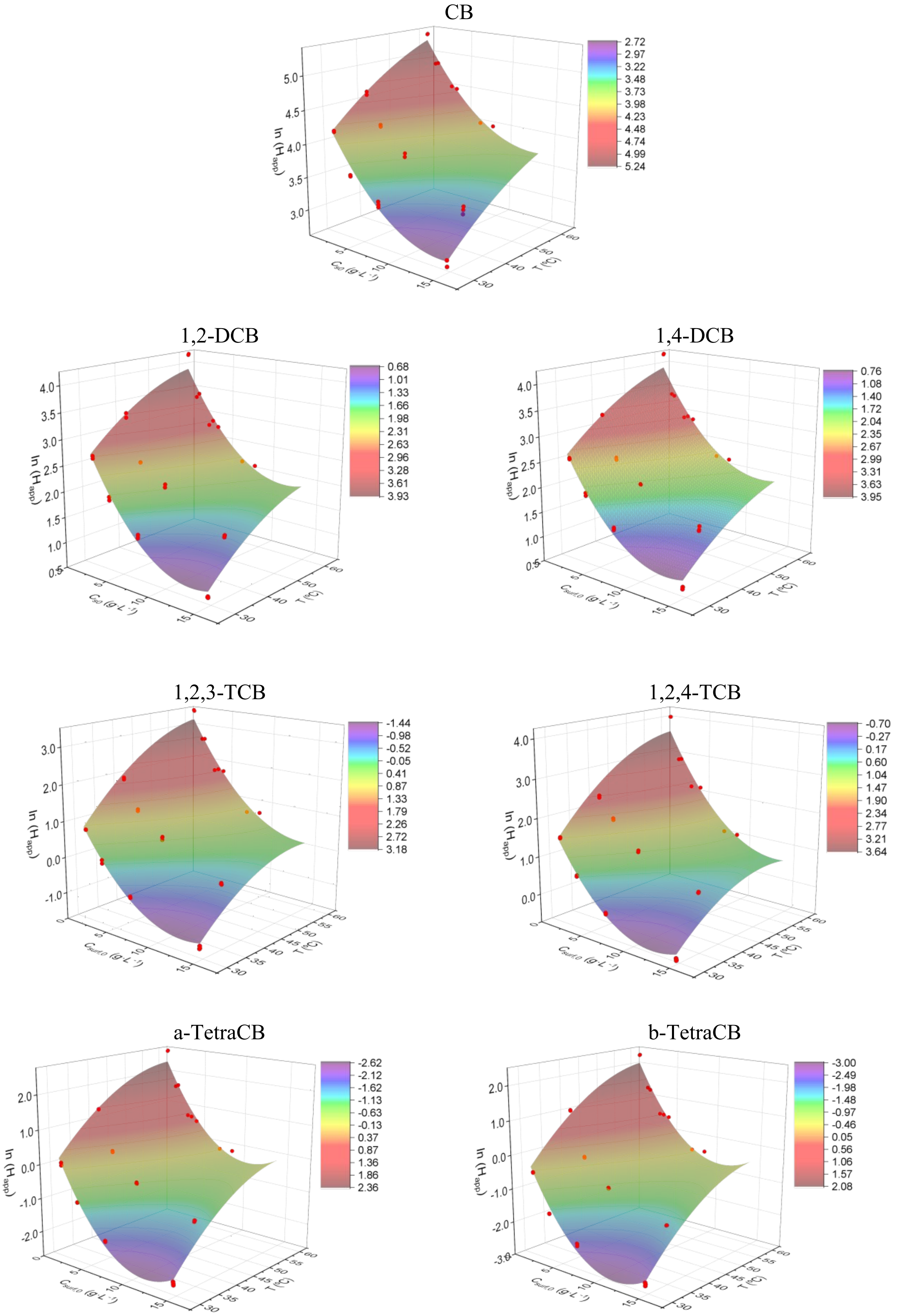

Figure S8.Values of $H_{app,j}$ for the experiments summarized in Table 1 set B2 (red points) and the response surfaces for the different compounds

Table S4. Surfactant concentration recalculated with the kinetic model proposed (Eq. (3)) considering the COCs concentration and temperature at equilibrium (1h).

$C_{S0}(g \cdot L^{-1})$ **20 ºC**	$C_{COCs}(mmol \cdot L^{-1})$	$C_{S0}(g \cdot L^{-1})$ **30 ºC**	$C_{S0}(g \cdot L^{-1})$ **40 ºC**	$C_{S0}(g \cdot L^{-1})$ **60 ºC**
1.5	2.3	1.4	1.3	1.2
1.5	4.7	1.5	1.3	1.1
3.5	5.9	3.4	3.0	2.6
3.5	14.6	3.4	3.0	2.2
7	5.9	6.8	6.0	5.0
7	17.6	6.8	6.0	4.3
7	29.3	6.8	6.0	3.8
15	11.7	14.6	12.9	10.1
15	23.4	14.7	12.8	9.4
15	46.9	14.7	12.8	7.9
